# Mushroom-derived polysaccharides in cancer immunotherapy: mechanisms, human evidence, and translational priorities

**DOI:** 10.3389/fonc.2026.1955408

**Published:** 2026-09-30

**Authors:** Sylvester Chibueze Izah, Ebinyo Rebecca Aseibai, Kingsley Excel Dunga, Matthew Chidozie Ogwu, Vivian Ibienebakabobo Promise, Nsikak Godwin Etim, Irene Nneka Ohanusi

**Affiliations:** 1 Department of Community Medicine, Faculty of Clinical Sciences, Bayelsa Medical University, Yenagoa, Bayelsa State, Nigeria; 2 Department of Microbiology, Faculty of Science, Bayelsa Medical University, Yenagoa, Bayelsa State, Nigeria; 3 Department of Microbiology, Federal University Otuoke, Bayelsa State, Nigeria; 4 Department of Medical Laboratory Science, Kingsley Ozumba Mbadiwe University, Ogboko, Imo State, Nigeria; 5 Goodnight Family Department of Sustainable Development, Appalachian State University, Boone, NC, United States; 6 Department of Public Health, Faculty of Health Sciences, Bayelsa Medical University, Yenagoa, Bayelsa State, Nigeria; 7 Niger-Delta Institute for Emerging and Re-emerging Infectious Diseases, Federal University Otuoke, Yenagoa, Bayelsa State, Nigeria; 8 Department of Microbiology, Faculty of Natural Sciences, Hezekiah University, Nkwerre, Imo State, Nigeria

**Keywords:** beta-glucan, cancer immunotherapy, dectin-1, mushroom polysaccharides, myeloid-derived suppressor cells, PSK, translational oncology, tumor microenvironment

## Abstract

Mushroom-derived polysaccharides are widely promoted as anticancer immune modulators, but interpretation is weakened by imprecise product identity, chemically non-equivalent preparations, and extrapolation across evidence levels. This critical narrative review evaluates beta-glucans, heteropolysaccharides, polysaccharide-protein complexes, crude fractions, modified derivatives, and multicomponent extracts as preparation-specific adjunct candidates. Receptor-level evidence is strongest for general fungal beta-glucan systems, particularly Dectin-1/Syk/CARD9 signaling and conditional complement receptor 3 cooperation; direct transfer of those mechanisms to individual mushroom preparations requires product-specific causal testing. Selected animal studies support myeloid remodeling, dendritic-cell and natural-killer-cell effects, and T-cell activation, but route, exposure, product characterization, and model limitations constrain translation. Human evidence remains heterogeneous: historical randomized PSK studies reported benefit in specific adjuvant chemotherapy settings, a later phase III lentinan trial was negative for overall survival, and small maitake, *Agaricus*, and pleuran studies mainly report immune surrogates. Direct clinical evidence with checkpoint inhibitors, therapeutic antibodies, or cancer vaccines is not established. The field should therefore move from species-level claims to product-level development with authenticated source material, orthogonal structural analysis, contaminant control, mechanism-linked potency, route-specific pharmacokinetics, tumor pharmacodynamics, and randomized testing against contemporary standard care. Mushroom-derived polysaccharide immunomodulators remain plausible research candidates, but neither the class nor unstandardized mushroom foods or supplements are clinically validated substitutes for established cancer treatment.

## 1 Introduction

Cancer remains a major global health burden, with an estimated 20.6 million new diagnoses and 9.8 million deaths in 2024 (1). Its genetic, metabolic, and immune heterogeneity limits the predictive value of isolated cytotoxicity assays and creates a need for adjunctive strategies that can be tested within a clearly defined tumor and treatment context (2).

Cancer immunotherapy has altered outcomes in several malignancies, but durable responses remain restricted to subsets of patients. Tumor-intrinsic signaling can exclude effector lymphocytes from the tumor microenvironment, and acquired resistance may emerge through defects in antigen presentation or interferon signaling (3, 4). Tumors may also recruit suppressive macrophages and myeloid-derived suppressor cells, accumulate regulatory T cells, and generate hypoxic and nutrient-limited conditions that impair immune function. These limitations support investigation of adjuncts that activate innate sensing, improve antigen presentation, reprogram suppressive myeloid cells, or strengthen antibody- and T-cell-mediated killing without producing uncontrolled systemic inflammation.

Mushrooms are fungal sources of chemically diverse macromolecules. Their polysaccharides include beta-glucans, alpha-glucans, mannans, galactans, mixed heteropolysaccharides, and protein-bound carbohydrate complexes. Lentinan from *Lentinula edodes*, pleuran from *Pleurotus ostreatus*, maitake fractions from *Grifola frondosa*, and protein-bound polysaccharide K (PSK) from *Trametes versicolor* are among the best known. However, a mushroom species is not a drug identity. Fruiting-body powders, mycelial biomass, hot-water extracts, alkali-insoluble glucans, protein-bound complexes, fermented preparations, and chemically modified derivatives are different interventions. Their activity may change with strain, substrate, harvest stage, extraction temperature, pH, molecular-mass distribution, branching, conformation, aggregation, and contaminant burden (5–7).

This Review uses the clearer term mushroom-derived polysaccharide immunomodulators and evaluates them as preparation-specific adjunct candidates rather than as living microbial therapies. Their most defensible investigational roles are as defined biological-response modifiers, receptor-targeted materials, adjuvants, or delivery platforms used with established anticancer treatment. Dietary mushroom consumption and administration of a structurally defined pharmaceutical preparation are not equivalent interventions.

Recent reviews have synthesized anticancer compounds from edible and medicinal mushrooms, beta-glucan immunobiology, clinical trials, and structure-function relationships (6–10). These reviews provide broad coverage, but their scopes do not consistently integrate product identity, causal evidence level, numerical human outcomes, negative trials, and a route-specific development framework in one preparation-centered analysis.

A systematic review of edible-fungal polysaccharide immunomodulation linked molecular mass, branching, conformation, and chemical modification with macrophage, dendritic-cell, and lymphocyte responses, while emphasizing species-specific recognition and the absence of standardized preparation protocols (11). A contemporary review of *Ganoderma lucidum* polysaccharides similarly found that extraction and modification alter composition and that structure-activity relationships remain incompletely resolved (12). Reviews focused on lentinan delivery and on mushroom clinical trials describe expanding formulation research but persistent heterogeneity in products, doses, endpoints, and clinical settings (13, 14). These publications update the evidence base without converting preclinical activity or mixed clinical signals into a class-wide efficacy claim.

The novelty of the present review is therefore fourfold: it treats the tested preparation, rather than the mushroom species, as the unit of inference; operationalizes an evidence hierarchy from material identity through receptor causality, animal tumor effects, human immune modulation, and clinical efficacy; integrates positive and negative human evidence using numerical outcomes and study limitations; and separates evidence-based conclusions from an explicitly proposed translational roadmap. These elements are intended to prevent class-wide claims from being built on chemically or clinically non-equivalent interventions.

This review examines four related questions: which preparation-specific structural features determine biological activity; what original evidence supports receptor engagement and modulation of innate and adaptive antitumor immunity; what human studies demonstrate when numerical outcomes, comparators, and limitations are considered; and which evidence-informed development priorities could make future oncology studies reproducible and interpretable. Direct tumor-cell effects are distinguished from host-mediated immunomodulation, and immune-marker changes are distinguished from clinical efficacy.

## 2 Review approach and evidence prioritization

A structured critical narrative-review approach was used because the literature spans carbohydrate chemistry, fungal taxonomy, immunology, oncology, formulation science, and clinical trials, and because the tested materials are too heterogeneous to support pooled analysis as a single intervention. PubMed, publisher databases, DOI records, and backward citation tracing were searched through 10 September 2026. Search terms combined common and scientific mushroom names with polysaccharide, beta-glucan, lentinan, pleuran, PSK, PSP, maitake, D-fraction, Dectin-1, complement receptor 3, macrophage, tumor-associated macrophage, dendritic cell, natural killer cell, myeloid-derived suppressor cell, T cell, checkpoint blockade, cancer vaccine, microbiome, single-cell, spatial profiling, cancer, clinical trial, pharmacokinetics, nanoparticle, and standardization.

Original studies were prioritized for preparation-specific claims. Eligible evidence included structural characterization, receptor and signaling experiments, immune-cell assays, immunocompetent animal tumor models, human immune studies, randomized or controlled oncology studies, pharmacokinetic investigations, and formulation or manufacturing studies. Preparation-specific preclinical studies were selected for comparative tabulation when they reported a preparation identity, model or cellular context, dose or exposure, comparator, and tumor or immune outcome. Recent evidence was included when it addressed myeloid suppression, T-cell responses, checkpoint combinations, microbiome-linked tumorigenesis, delivery, or modern immune profiling. Studies lacking adequate preparation description were retained only when historically influential and are labeled accordingly. Review articles were used for contextual synthesis or identification of primary studies. Commercial webpages and claims that could not be traced to peer-reviewed literature were excluded. Historical taxonomic names were retained when required to identify the tested preparation and were linked to accepted species names where possible, particularly for *Agaricus subrufescens*, *A. blazei*, and *A. brasiliensis* (15).

For each study, the review considered the source tissue or culture material, extraction and purification method, structural definition, dose, route, model, comparator, immune endpoint, tumor endpoint, adverse events, and principal limitation. Human studies were interpreted according to randomization, sample size, product identity, standard-care control, duration, and whether the endpoint was survival, recurrence, objective response, quality of life, or an immune biomarker. Cell-culture inhibition was not labeled clinical antitumor efficacy, animal tumor suppression was not described as a patient benefit, and an increase in cytokines or lymphocyte counts was not treated as proof of tumor control.

Because this article is a narrative review rather than a systematic review, PRISMA-based study selection and a uniform risk-of-bias instrument were not applied across the chemically and methodologically diverse evidence base. Non-indexed and non-English publications may therefore be underrepresented. The synthesis emphasizes preparation-specific evidence, verifies influential quantitative findings, includes negative results, and identifies the translational conditions required for interpretable future studies.

## 3 Evidence hierarchy and claim discipline

A central weakness in this literature is the collapse of unlike evidence into one narrative. A chemically characterized glucan, a crude mushroom extract, an immune-cell assay, an animal tumor experiment, and a randomized clinical trial answer different questions. The strongest review therefore begins with an evidence hierarchy rather than with a list of species. At the foundation is material identity: the species or strain, tissue or culture source, extraction process, molecular-mass distribution, linkage pattern, purity, and formulation must be sufficiently described to know what was tested. Without this foundation, even an apparently rigorous biological experiment may be irreproducible because another laboratory cannot obtain an equivalent intervention.

Mechanistic evidence occupies the next level. Receptor binding is informative only when productive signaling and functional consequences are demonstrated. For example, Dectin-1 engagement becomes a causal mechanism when receptor blockade, deficient cells, or downstream Syk/CARD9 disruption reduces the observed response. A rise in nuclear factor-kappaB activity alone is less specific because endotoxin, nucleic acids, proteins, or other co-extracted ligands can produce similar inflammatory signals. Likewise, macrophage polarization described through one or two markers is not equivalent to functional reprogramming. The stronger experiment links receptor dependence to phagocytosis, antigen presentation, T-cell priming, tumor-cell killing, or another prespecified function (16–19).

Animal evidence should then show that the proposed mechanism operates in a relevant tumor system. An intervention given before or immediately after implantation tests prevention or early establishment, not treatment of a clinically established tumor. Subcutaneous transplant models are useful for screening but may not reproduce organ-specific stroma, metastatic spread, treatment history, or the immune architecture of human disease. Dose, route, timing, sex, age, microbiota, and the use of immunocompetent versus immunodeficient animals all influence interpretation. Stronger studies include a contemporary comparator, test more than one model, measure exposure, and use depletion, knockout, or adoptive-transfer experiments to show that tumor control is lost when the claimed immune pathway is removed (20).

Human evidence requires a further distinction between biological activity and clinical utility. A controlled increase in natural-killer-cell activity, CD8-positive cells, cytokines, or gene expression may support a pharmacodynamic effect, but it does not establish that patients live longer, remain recurrence-free, experience tumor regression, or tolerate standard therapy better. Small exploratory studies can identify a safe dose and a biomarker, but multiple immune outcomes create a high risk of chance findings unless the analysis is prespecified. Clinical efficacy is most credible when a randomized study uses a defined product, balances prognostic factors, applies current standard care, reports adverse events and attrition, and measures a patient-centered endpoint (21).

This hierarchy also changes how negative evidence is interpreted. A negative trial does not always invalidate the biological mechanism. Failure may arise because the tested product lacked the intended structure, oral delivery produced insufficient exposure, the selected cancer lacked the receptor or immune context, or the partner therapy did not create the required complement or antigen-presentation signal. Conversely, a positive trial cannot rescue an undefined product. If the preparation cannot be reproduced, the result remains historically interesting but difficult to translate.

The review uses explicit claim labels. Demonstrated denotes a mechanism established by direct binding plus productive signaling and causal interference, or a numerical clinical outcome from an adequately described controlled trial; the label is confined to the material and system actually tested. Supported denotes consistent preparation-specific findings with important limitations. Hypothesized denotes a biologically plausible pathway that has not been causally shown for the named mushroom preparation or in the relevant clinical setting. Preliminary applies to small, uncontrolled, or surrogate-endpoint studies, and inconsistent denotes conflicting findings across comparable experiments or trials. These labels prevent the common progression from a general fungal beta-glucan mechanism to a class-wide mushroom efficacy claim.

The same discipline applies to novelty. Adding another species with total-polysaccharide yield, antioxidant activity, and one tumor-cell line contributes little to oncology unless it resolves a mechanistic or translational question. Higher-value studies compare defined structural variants, explain why activity differs, measure tumor delivery, identify a responsive immune compartment, or test a rational combination. In this field, negative and comparative data may be more informative than another positive extract screen because they establish boundaries of activity and help select a product that can survive clinical development.

Evidence should also be interpreted through the intended use. A candidate proposed as an anticancer drug must ultimately improve tumor control or survival. A supportive-care product may instead aim to reduce infection, maintain chemotherapy dose intensity, or improve validated symptoms, but it should not be described as tumor-directed immunotherapy unless tumor outcomes are tested. A delivery material may be valuable because it transports antigen or another drug, even when the glucan itself has little independent efficacy. Defining the intended role in advance prevents endpoints from being changed after results are known. It also determines which comparator, dose, safety tests, and regulatory pathway are appropriate.

Statistical significance should not be equated with evidential strength. Small studies can produce low p values for selected biomarkers while providing imprecise estimates and limited external validity. Effect sizes, confidence intervals, absolute event rates, missing data, and the number of outcomes examined are more informative than isolated significance statements. These details are reported when they were available in the original publications; absent information is identified as a limitation (21).

## 4 Product identity, extraction, and structure-activity relationships

The first translational requirement is material identity. Terms such as “mushroom beta-glucan,” “total polysaccharide,” or “D-fraction” may conceal substantial chemical variation. Fungal beta-glucans commonly contain a beta-(1,3)-linked glucose backbone with beta-(1,6) branches, but backbone length, branch frequency, side-chain length, molecular mass, conformation, solubility, and particle size vary among preparations. Heteropolysaccharides may also contain mannose, galactose, xylose, fucose, arabinose, or uronic acids, and protein-bound complexes may display properties that cannot be assigned to carbohydrate alone (22–24).

Historical work illustrates why preparation identity matters. Chihara et al. (22) isolated antitumor polysaccharides from *L. edodes* and established the experimental basis for lentinan development. Lentinan is usually described as a purified, branched beta-(1,3)-D-glucan with beta-(1,6) side chains, but clinical conclusions apply to specified formulations and routes, not to shiitake food. Karacsonyi and Kuniak (23) characterized pleuran from *P. ostreatus* as an alkali-insoluble branched beta-D-glucan. Later use of the word pleuran without demonstrating chemical equivalence can therefore be misleading. Nanba et al. (24) characterized a maitake antitumor polysaccharide, while subsequent D- and MD-fraction labels became process-dependent commercial descriptors rather than universal molecular entities. PSK is still more distinct: it is a protein-bound polysaccharide complex obtained from cultured *T. versicolor* mycelium and cannot be treated as chemically interchangeable with purified lentinan.

Extraction changes the candidate. Hot water may recover soluble polysaccharides but can reduce molecular mass during prolonged heating. Alkali can release cell-wall glucans while altering conformation or substitution. Enzymes, ultrasound, microwave treatment, or subcritical water can increase yield yet change the distribution of fragments and aggregates. Ethanol precipitation enriches high-molecular-weight carbohydrate but does not create a homogeneous product. Crude preparations can retain proteins, pigments, phenolics, nucleic acids, small carbohydrates, minerals, bacterial endotoxin, or substrate-derived starch. In macrophage assays, endotoxin or other co-extracted Toll-like-receptor agonists can be mistaken for polysaccharide-mediated inflammatory activity. Endotoxin should be quantified, and orthogonal controls such as receptor-deficient cells or validated neutralization controls should be used where appropriate (25–27).

Purification usually combines dialysis or ultrafiltration with ion-exchange and size-exclusion chromatography. Each fraction should be evaluated using orthogonal methods: monosaccharide composition, methylation or linkage analysis, nuclear magnetic resonance, molecular-mass distribution, protein and nucleic-acid content, residual solvent testing, and conformation-sensitive methods where relevant. Analytical measurements should be performed under formulation-relevant conditions because a triple helix measured in one solvent may become a random coil or aggregate after sterilization, dilution, storage, or contact with plasma proteins (5, 7, 26).

Source control begins before extraction. Species and strain should be authenticated, the fruiting body or mycelial material identified, and the cultivation substrate, harvest stage, drying method, and storage conditions reported. Mycelial products grown on grain are particularly vulnerable to residual substrate carbohydrate being counted as fungal polysaccharide. Food-grade production is not sufficient for parenteral or pharmaceutical use: release specifications must address bioburden, endotoxin, mycotoxins, heavy metals, pesticide residues, protein impurities, batch fingerprints, and a mechanism-linked potency assay. A credible potency assay might quantify Dectin-1-dependent reporter activation, receptor-blockable macrophage phagocytosis, or dendritic-cell costimulation, provided that the assay does not respond equally to endotoxin and unrelated inflammatory contaminants. **Table 1** applies these principles to representative mushroom materials. The purpose is to show that evidence belongs to a defined preparation (7, 26, 27).

**Table 1:** Representative mushroom polysaccharide preparations and evidence.

Source species	Intervention class	Principal preparation	Evidence base	Evidence-based interpretation	References
*Lentinula edodes*	Purified beta-glucan	Lentinan; branched beta-(1,3)/(1,6)-glucan	Foundational mouse studies; older gastric-cancer literature; negative phase III trial	Formulation- and regimen-specific; not equivalent to shiitake food	22, 28
*Trametes versicolor*	Polysaccharide-protein complex	PSK from cultured mycelium	Randomized adjuvant gastric and colorectal trials	Historically important clinical evidence; contemporary replication needed	29, 30
*Grifola frondosa*	Purified fraction or crude/process-defined fraction	Maitake fractions, including heterogeneous GFI	Structure studies; 4T1 myeloid-remodeling model; small human dose escalation	Preparation-specific animal support; human efficacy unproven	24, 31, 32
*Ganoderma lucidum*	Purified/characterized polysaccharide; spore fraction; conjugate	GLP preparations and engineered derivatives	LLC MDSC model; colorectal anti-PD-1 mouse study; microbiome/tumorigenesis and delivery studies	Recent preclinical support; cross-study equivalence and clinical benefit unproven	33–36
*Pleurotus ostreatus*	Purified beta-glucan or process-defined supplement	Pleuran and related beta-glucans	Defined pleuran structure; nonrandomized human immune study	Immune monitoring feasible; recurrence or survival benefit unestablished	23, 37
*Agaricus subrufescens* complex	Multicomponent extract or crude fraction	Products historically labeled *A. blazei*/*A. brasiliensis*	Gynecologic-cancer and multiple-myeloma immune studies	Immune-marker effects; taxonomy and product heterogeneity limit inference	38, 39
*Hericium erinaceus*	Heteropolysaccharide or modified derivative	Native and hydroxyethylated polysaccharides	Structural and macrophage studies	Useful for structure-activity research; no validated clinical oncology role	40, 41
*Flammulina velutipes*	Purified heteropolysaccharide	FVPA1 and other characterized fractions	NK-cell and B-lymphocyte studies	Mechanistic *in-vitro* evidence; no controlled cancer trial	42, 43
*Auricularia* species	Crude fraction or modified derivative	Crude, native, and periodate-modified polysaccharides	Macrophage-conditioned cell study and early animal work	Immune-mediated concept supported; clinical evidence absent	44, 45
*Inonotus* species	Acidic/neutral heteropolysaccharides or polysaccharide-protein complexes	IOP-1, ESP/DSP, and PSP-1	Recent lung- and liver-cancer cell and animal studies	Direct antitumor activity reported; immune causality and clinical efficacy remain unestablished	46–48

Evidence applies to the named preparation and setting, not to all materials from the species. GLP, *Ganoderma lucidum* polysaccharide; MDSC, myeloid-derived suppressor cell; NK, natural killer; PSK, polysaccharide K; PSP, polysaccharide-protein complex.

Structure-activity relationships are multivariable rather than governed by a single optimal molecular mass. In general fungal beta-glucan systems, particulate presentation, higher-order structure, and epitope exposure influence Dectin-1 clustering and signaling, whereas soluble ligands may bind without creating the same spatial signal (19, 49). These findings demonstrate a general biophysical principle, not the potency of every mushroom beta-glucan. Large polymers may increase multivalent presentation but can have poorer conventional absorption and greater aggregation. Chemical modification can increase solubility or charge, yet the derivative becomes a new entity whose receptor profile, pharmacokinetics, and safety must be re-established. Ren et al. (40), for example, reported greater macrophage-stimulating activity after hydroxyethylation of a *Hericium erinaceus* polysaccharide; that result supports engineering but cannot establish identical effects for the native dietary polymer. **Table 2** compares the principal structural variables with the evidence actually available.

**Table 2:** Structural variables and preparation-specific biological evidence.

Structural variable	Comparative preparation evidence	Receptor/immune association	Dose, model, and quantitative outcome (units as reported)	Evidence interpretation	References
Molecular-mass distribution	GFI: broad 0.24-99.9 × 10^4^ Da; GLP: reported average 4.44 × 10^4^ Da; WEP1: 662.1 kDa	Mass may affect multivalency, solubility, aggregation, and exposure; no universal optimum	GFI 25–100 mg/kg i.p., 4T1: dose-related tumor reduction and MDSC changes; WEP1 50–200 mg/kg, H22: direct antitumor effects	Supported within each preparation; cross-product comparisons are confounded by route and composition	32, 33, 48, 50–53
Backbone linkage and branching	Lentinan and pleuran are branched beta-(1,3)/(1,6)-glucans; heteropolysaccharides contain additional sugars/linkages	Beta-glucan epitopes can support Dectin-1 recognition; branch architecture and epitope exposure affect presentation	Foundational sarcoma-180 activity for lentinan; historical structure/activity work for pleuran and maitake	Structure is documented, but receptor causality and clinical transfer remain preparation-specific	11, 12, 22–24, 41, 43
Conformation and particulate presentation	Triple-helix, coil, and particulate states can change with solvent, processing, storage, and formulation	Particulate or exposed beta-glucan supports Dectin-1 clustering and phagocytic-synapse signaling	General fungal beta-glucan systems show signaling dependence on presentation; direct tumor-dose comparisons across mushroom products are sparse	Demonstrated general biophysical principle; often hypothesized rather than measured for named mushroom products	19, 49
Solubility and aggregation	Hot-water, alkali, fragmentation, and formulation alter soluble/insoluble fractions and aggregate size	Solubility changes receptor access and biodistribution; binding alone may not yield productive signaling	Route-dependent exposure is rarely measured; oral and parenteral results cannot be assumed equivalent	Major translational gap; requires formulation-state and exposure measurements	5, 49, 54
Protein association and purity	PSK is a polysaccharide-protein complex; crude *Agaricus* and *Auricularia* preparations contain multiple constituents	Observed activity cannot be assigned to carbohydrate without fractionation; endotoxin or co-extracted agonists can confound inflammatory assays	Historical PSK clinical outcomes apply to the specified complex; crude-extract immune effects remain attribution-limited	Product identity and contaminant controls determine causal confidence	25, 29, 30, 38, 45
Chemical modification or conjugation	Hydroxyethylated *Hericium* derivative; periodate-modified *Auricularia* glucan; *Ganoderma* polysaccharide-methotrexate conjugate	Modification may alter charge, solubility, receptor clustering, uptake, release, and safety	Hydroxyethylated derivative increased RAW264.7 activity; engineered *Ganoderma* conjugate supported preclinical drug delivery	A derivative is a new product; native-polymer efficacy cannot be inferred	13, 35, 40, 44, 55, 56

Da, dalton; GFI, *Grifola frondosa* polysaccharide-rich fraction; GLP, *Ganoderma lucidum* polysaccharide; i.p., intraperitoneal; MDSC, myeloid-derived suppressor cell. Molecular masses, dose ranges, and effect estimates are reported in the units used by the cited studies.

Recent quantitative studies illustrate why molecular description must remain attached to biological findings. Feng et al. (51) isolated the 6.4-kDa *Ganoderma sinense* polysaccharide GSP70-2, composed of glucose, mannose, and N-acetylglucosamine in a molar ratio of 4.4:6.1:1.0, after screening crude fractions in a 4T1 model. He et al. (52) characterized *Trametes orientalis* TOP60–1 as a homogeneous 1.122 × 10^4^-g/mol heteropolysaccharide and reported dose-dependent tumor, migration, angiogenesis, and macrophage-polarization effects in zebrafish and cell assays. Guo et al. (48) characterized an *Inonotus hispidus* polysaccharide-protein complex with an average molecular mass of 327 kDa, 64.48% carbohydrate, and 28.89% protein; at 100 mg/kg it produced 36.9 ± 2.3% tumor inhibition in a hepatocellular-carcinoma model, while the reported HepG2 half-maximal inhibitory concentration was 87.9 micrograms/mL. These results are preparation-specific and predominantly preclinical; they do not establish interchangeable activity or human efficacy.

## 5 Immune recognition and antitumor mechanisms

Dectin-1 is the most established receptor in general fungal beta-glucan biology, but the foundational causal experiments did not test all mushroom preparations reviewed here. Brown et al. (16) used receptor-specific inhibition and an anti-Dectin-1 antibody to show that Dectin-1 was the predominant receptor for beta-glucan-dependent, non-opsonic recognition of zymosan by primary macrophages. Taylor et al. (57) mapped strong expression to monocyte/macrophage and neutrophil lineages. Ligand-induced receptor clustering recruits spleen tyrosine kinase (Syk), and Syk-dependent signaling can drive cytokine production independently of or cooperatively with Toll-like receptors (17). CARD9 links Dectin-1 signaling to BCL10/MALT1 and nuclear factor-kappaB; CARD9-deficient systems established this pathway in antifungal immunity (18). Accordingly, Dectin-1/Syk/CARD9 is demonstrated for defined general fungal beta-glucan systems but remains supported or hypothesized for a named mushroom preparation unless receptor-dependent experiments were performed with that exact material.

Physical presentation is as important as nominal receptor affinity. Goodridge et al. (19) showed in a general particulate beta-glucan system that Dectin-1 activation is favored when a receptor-rich contact zone excludes inhibitory phosphatases; Anaya et al. (49) further linked fungal beta-glucan structure and exposure to Dectin-1 multimerization and signaling. These demonstrated biophysical observations explain why soluble and particulate glucans with similar linkages may produce different responses. They do not, by themselves, demonstrate target engagement by a mushroom extract. Preparation-specific studies should therefore show receptor dependence, exclude endotoxin and other co-extracted agonists, and preserve the relevant conformation under assay and formulation conditions.

Complement receptor 3 (CR3; CD11b/CD18) provides a second demonstrated but conditional mechanism in general beta-glucan systems. Hong et al. (58) showed that beta-glucan could support monoclonal-antibody therapy by priming CR3-positive granulocytes to kill iC3b-coated tumor cells. The mechanism requires complement deposition, a compatible glucan fragment, CR3-expressing effector cells, and access to the target; it is not established for all mushroom preparations. Proposed cooperation with mannose, scavenger, or Toll-like receptors is likewise hypothesized until receptor dependence is demonstrated by blocking, genetic loss, competition, direct binding, or rescue experiments with the preparation under study.

Macrophages and suppressive myeloid cells are major preparation-specific targets because they express glucan-recognition receptors and populate many tumors. The conventional M1/M2 vocabulary is useful for orientation but oversimplifies the multidimensional states found in human tumors. Wang et al. (59) reported that a mushroom beta-glucan altered tumor-associated macrophage features in a Lewis lung carcinoma model. In a separate Lewis lung carcinoma study, a characterized *Ganoderma lucidum* polysaccharide reduced myeloid-derived suppressor cells and was associated with CARD9/NF-kappaB/IDO signaling, increased CD4- and CD8-positive T cells, and tumor inhibition (33). Nilkhet et al. (45) used conditioned media from *Auricularia polytricha* crude-polysaccharide-polarized macrophages and observed reduced invasion and increased apoptosis in breast-cancer cell models. Wold et al. (25) showed that purified acidic, water-soluble *Inonotus obliquus* fractions AcF1 and AcF3 activated TLR2 and TLR4 reporter systems and weakly activated Dectin-1; with interferon-gamma, they induced tumoricidal macrophage activity. The *in-vivo* component co-injected preactivated macrophages with tumor cells and therefore did not establish systemic polysaccharide exposure or treatment of an established tumor. Collectively, these studies support preparation-specific myeloid remodeling, but causal pathways, product comparability, systemic exposure, and human tumor confirmation remain incomplete.

Dendritic cells connect innate recognition to adaptive immunity. Dectin-1/Syk/CARD9 can promote dendritic-cell maturation and T-cell differentiation in general fungal-recognition systems (60). For a mushroom preparation, receptor-dependent dendritic-cell activation is supported only when the exact product is tested and a causal intervention is included. In oncology, stronger evidence would show presentation of a defined tumor antigen, expansion of antigen-specific CD8-positive T cells, and loss of tumor control after dendritic-cell or T-cell depletion. Surface CD80 or CD86 expression alone is insufficient to demonstrate functional antitumor priming.

Natural killer (NK) cells may respond directly or indirectly. Jia et al. (42) characterized the *F. velutipes* polysaccharide FVPA1 and reported enhanced NK-cell activity against K562 cells *in vitro*. Ahn et al. (38) observed higher NK activity in patients receiving an *Agaricus* extract during chemotherapy. These findings do not establish a shared receptor mechanism on NK cells; macrophage- or dendritic-cell cytokines such as interleukin-12, interleukin-15, and type I interferons may mediate the effect. Tumor trafficking, persistence, and exhaustion must also be measured because increased cytotoxicity in peripheral blood does not guarantee activity inside the tumor.

Adaptive antitumor effects are expected to be indirect unless a polysaccharide is engineered to carry tumor antigen. Improved antigen presentation, inflammatory context, chemokine production, and myeloid reprogramming can support CD4-positive and CD8-positive T-cell responses. A 2024 colorectal-cancer mouse study reported that a *Ganoderma lucidum* polysaccharide increased T-cell-mediated antitumor immunity and improved the activity of anti-PD-1 treatment (36). This is preparation-specific preclinical support, not clinical validation. Mushroom preparations are therefore plausible partners for checkpoint blockade, antibodies, vaccines, radiotherapy, or immunogenic chemotherapy, but each combination requires direct tests of product identity, schedule, target engagement, interaction, and toxicity.

Immune activation can also be harmful because the consequences of inflammatory signaling depend on dose, timing, tissue compartment, tumor context, and concomitant treatment. Dose selection should seek a reproducible, compartment-appropriate pharmacodynamic effect rather than the largest cytokine increase, and time-course and repeat-dose studies are needed to detect desensitization, rebound suppression, or clinically relevant toxicity (2, 27).

## 6 Tumor-microenvironment and preclinical evidence

Preparation-specific animal studies show that some mushroom polysaccharides can influence tumor growth, but they also expose the gap between experimental efficacy and clinical readiness. Chihara et al. (22) reported inhibition of sarcoma 180 by *L. edodes* polysaccharides, and early work with maitake and *Auricularia* established that antitumor activity depended on structure and chemical modification (24, 44). These studies were foundational, but many used transplantable tumors, early treatment, high doses, or endpoints that do not model contemporary metastatic disease.

A recent *Grifola* study provides a more informative immune chain but also illustrates why chemical terminology matters. Li et al. (32) prepared GFI from fruiting bodies by hot-water extraction, ethanol precipitation, dialysis, and freeze-drying and reported a broad molecular-mass range (0.24-99.9 × 10^4^ Da). GFI should therefore be described as a heterogeneous polysaccharide-rich fraction, not a chemically defined purified polysaccharide. In female immunocompetent BALB/c mice bearing established 4T1 tumors, intraperitoneal GFI at 25, 50, or 100 mg/kg every other day reduced tumor weight and decreased polymorphonuclear myeloid-derived suppressor cells while strengthening T-cell-associated responses. Tumor endpoints used 10 mice per group, whereas immune-assay sample sizes varied by endpoint. The absence of direct 4T1 cytotoxicity supported a host-mediated interpretation, but receptor causality, route-specific exposure, and human-cell bridging remain unresolved.

Direct tumor-cell activity should be presented separately. Zhang et al. (50) purified a 662.1-kDa water-extracted *L. edodes* polysaccharide, WEP1, and tested it in H22-bearing mice at 50, 100, and 200 mg/kg. Tumor-inhibition rates were 28.27%, 35.17%, and 51.72%, respectively. The study linked activity to reactive oxygen species, mitochondrial apoptosis, G2/M arrest, and inhibition of tubulin polymerization. These results support antitumor potential, but the proposed mechanism is principally direct cytotoxicity rather than immune modulation. Moreover, the relation between the administered dose, systemic exposure, and concentrations used *in vitro* was not fully resolved. Including such studies is useful only when the mechanistic category is explicit.

Other studies offer narrower but relevant signals. A characterized *Ganoderma lucidum* polysaccharide reduced myeloid-derived suppressor cells in a Lewis lung carcinoma model and implicated CARD9-NF-kappaB-IDO signaling (33). Smiderle et al. (61) reported structural similarities and immunomodulatory activity for *Agaricus* polysaccharides in THP-1 cells; Ren et al. (40) showed that hydroxyethylation altered *Hericium erinaceus* polysaccharide activity in RAW264.7 macrophages; Yang et al. (41) combined structural characterization with immune assays; and Wang et al. (43) characterized a *Flammulina velutipes* polysaccharide with B-lymphocyte effects. Collectively, these studies support biological diversity, not equivalence across preparations from the same species.

Recent studies have shown direct-antitumor evidence but do not remove its translational limitations. *Ganoderma sinense* GSP70–2 suppressed 4T1-cell proliferation and altered apoptosis-related measures after structural definition (51). *Inonotus obliquus* IOP-1 inhibited NCI-H460 proliferation and migration and suppressed xenograft growth, whereas fractionated *Inonotus sanghuang* polysaccharides inhibited HepG2 and A549 growth and migration and suppressed corresponding xenografts without evident major-organ toxicity in the reported experiments (46, 47). These studies used cell lines and animal models, and their findings should be interpreted as preparation-specific preclinical activity rather than evidence of clinical immunotherapy benefit.

The tumor microenvironment should be measured directly. Peripheral cytokines or spleen-cell activity do not show whether a candidate reaches the tumor, reduces suppressive myeloid cells, improves dendritic-cell function, or expands antigen-specific T cells. Stronger studies should use immunocompetent orthotopic or genetically engineered models, established tumors, clinically relevant partners, and both sexes when biologically appropriate. Tumor tissue should be assessed by flow cytometry, spatial imaging, single-cell methods, or multiplex histology, while depletion or receptor-deficient experiments establish causality. Randomization, blinding where feasible, prespecified exclusions, and complete reporting of animal numbers and outcomes should follow established animal-research guidance (2, 20).

Combination experiments need a mechanistic reason. A recent *Ganoderma lucidum* polysaccharide study in mouse colorectal cancer supported improved T-cell-mediated activity with anti-PD-1 treatment (36), but direct clinical evidence for mushroom-derived preparations combined with checkpoint inhibitors, therapeutic antibodies, or cancer vaccines remains unestablished. A small single-arm clinical study of whole-glucan particles with PD-1/PD-L1 blockade used a yeast-derived product, not a mushroom preparation, and cannot be transferred across source or product identity (62). Similarly, beta-glucan/anti-PD-L1 activity in an *Aureobasidium* melanoma model is useful contextual evidence but is not mushroom-specific (63). Future combination studies should include each single agent, interaction and schedule analyses, causal immune-cell or receptor tests, blinded tumor assessment, and toxicity monitoring.

Recent combination studies strengthen the preclinical rationale while preserving the clinical boundary. In immunocompetent mouse models, lentinan at 1.25 mg/kg daily for 7 days was combined with 1 × 10^7^ CAR-T cells; groups contained nine mice, and the combination improved tumor control and survival across colon-carcinoma and melanoma models, with reported group comparisons at p < 0.001 (64). A *Ganoderma lucidum* polysaccharide-decorated extracellular-vesicle formulation administered intravenously at 90 mg/kg polysaccharide plus 10 mg/kg vesicles on days 6, 9, 12, 15, and 18 produced an 80.4% CT26 tumor-inhibition rate, compared with 62.6% for polysaccharide alone and 24.0% for vesicles alone (n = 7 per efficacy group); immune profiling used n = 3 and showed CD11b-positive/CD86-positive cells of 45.4% and CD3-positive/CD8-positive T cells of 14.0%, versus 20.2% and 1.9%, respectively, with PBS (56). A dendritic-cell-targeted full-cell nanovaccine assembled with *Ganoderma lucidum* polysaccharide also inhibited orthotopic, recurrent, and metastatic 4T1 tumors in mice (55). These engineered platforms are new products and require independent characterization, safety assessment, and clinical testing.

A recurrent flaw is the assumption that oral administration and parenteral administration test the same biological mechanism. High-molecular-weight glucans have limited conventional absorption. Oral activity may involve gut-associated immune sampling, microbial fermentation, barrier effects, or uptake of smaller fragments, whereas intravenous lentinan exposes blood and tissue phagocytes directly. Route-specific development is therefore necessary. Oral studies should measure the intact polymer or validated fragments, fecal recovery, microbiome changes, and systemic pharmacodynamic markers. Parenteral studies require sterility, endotoxin control, complement and infusion-reaction monitoring, tissue distribution, and clearance. Without these data, a negative outcome cannot distinguish biological failure from inadequate delivery, and a positive immune signal cannot be linked confidently to the proposed molecule. Representative preparation-specific preclinical studies are summarized in **Table 3** (6, 54).

**Table 3:** Preparation-specific preclinical evidence and causal limitations.

Preparation and characterization	Model, timing, dose, and route (mg/kg unless noted)	Comparator	Immune endpoints and causal tests	Tumor or functional outcome	Principal limitations	References
*G. frondosa* GFI; heterogeneous hot-water/ethanol-precipitated, dialyzed fraction; 0.24-99.9 × 10^4^ Da	Female BALB/c, immunocompetent 4T1; treatment from day 7; 25, 50, 100 mg/kg i.p. every other day	PBS/control; lentinan 50 mg/kg	Tumor/spleen PMN-MDSCs, CD8+ cells, granzyme B, TIGIT; no direct 4T1 cytotoxicity; receptor dependence not tested	Dose-related tumor-weight inhibition; immune endpoints supported host-mediated activity	Tumor n = 10/group; immune n varied; i.p. exposure not clinically bridged	32
*G. lucidum* GLP; homogeneous fraction, average molecular mass 4.44 × 10^4^ Da	Male C57BL/6, immunocompetent LLC; treatment after tumors reached about 50 mm³; 25 or 100 mg/kg by daily intragastric administration for 2 weeks	PBS vehicle; cisplatin positive control	MDSCs, CD4+/CD8+ T cells, IFN-gamma/IL-12; CARD9-NF-kappaB-IDO associations; full causal loss-of-function chain incomplete	Reduced tumor growth/weight and MDSCs	Single mouse model; systemic exposure and human equivalence unresolved	33
*G. lucidum* polysaccharide; preparation-specific characterization reported in source	Immunocompetent colorectal-cancer mouse models; GLP 300 mg/kg by oral gavage; anti-PD-1–100 micrograms/mouse	Vehicle, GLP, anti-PD-1, combination	T-cell profiling and functional antitumor immunity	Combination improved antitumor efficacy over monotherapy in the reported models	Preclinical; product equivalence, route-specific exposure, and clinical benefit unestablished	36
*G. lucidum* spore GLP; water-soluble fraction	AOM/DSS colitis-associated tumorigenesis; 200 or 300 mg/kg orally	AOM/DSS control and dose groups	Microbiota, short-chain fatty acids, barrier markers, macrophage/inflammatory signaling; microbiome necessity not proven by transfer/depletion	Reduced disease activity and colon tumor number/size	Prevention/inflammation model, not established-cancer immunotherapy	34
*L. edodes* WEP1; purified 662.1-kDa water-extracted polysaccharide	H22-bearing mice; 50, 100, 200 mg/kg	Tumor control and dose groups	ROS, mitochondrial apoptosis, G2/M arrest, tubulin polymerization; immune dependence not tested	Tumor inhibition 28.27%, 35.17%, 51.72%	Principally direct antitumor mechanism; exposure bridge incomplete	50
*A. polytricha* crude polysaccharides	THP-1-derived macrophage-conditioned MCF-7 and MDA-MB-231 models; *in-vitro* exposure	Untreated macrophage-conditioned controls	M1-associated markers, invasion and apoptosis; no receptor blockade	Conditioned responses reduced invasion and increased apoptosis	Crude composition; conditioned-media cell model; no *in-vivo* tumor confirmation	45
*I. obliquus* AcF1/AcF3; purified acidic water-soluble heteropolysaccharide fractions	Mouse and human macrophages; 1–100 micrograms/mL *in vitro*; BMDMs activated with 100 micrograms/mL plus IFN-gamma before LLC co-injection into syngeneic C57BL/6 mice	Untreated or IFN-gamma-only macrophages; Pam3CSK4 positive control; other fungal fractions	NO and cytokines; TLR2/TLR4/Dectin-1 reporters; TLR4-deficient cells and endotoxin controls	Macrophage-mediated LLC growth inhibition *in vitro*; only 2/9 mice in each AcF1/AcF3 group developed tumors after co-injection	Macrophage/tumor co-injection prevention design; no systemic polysaccharide dosing or established-tumor treatment	25
*F. velutipes* FVPA1; characterized heteropolysaccharide	NK-cell/K562 co-culture; *in vitro*	Relevant cell controls	NK-cell cytotoxic activity; receptor/cell-depletion causality not applicable or not shown	Enhanced NK activity against K562 cells	No tumor trafficking, persistence, or *in-vivo* efficacy	42
*H. erinaceus* hydroxyethylated derivative	RAW264.7 macrophages; *in vitro*	Native/modified or untreated comparisons	Nitric oxide and cytokine-related macrophage activity; no tumor model	Enhanced macrophage-associated activity after modification	Modified derivative is not equivalent to native dietary polymer	40
*L. edodes* lentinan (LTN); commercial beta-glucan preparation	C57BL/6J mice with EGFRvIII-positive or hCD19-positive MC38/B16F10 tumors; 1 × 10^7^ CAR-T cells i.v.; LTN 1.25 mg/kg i.p. daily for 7 days; n = 9/group	Untransduced T cells; CAR-T cells; LTN alone in a supplementary comparison	CAR-T memory/exhaustion markers, intratumoral cytokines, persistence, and macrophage markers; no receptor-loss test	Combination reduced tumor growth and prolonged survival across four model/target settings; reported group comparisons p < 0.001	Engineered murine targets and CAR-T cells; no human trial; product exposure not reported	64
*G. lucidum* polysaccharide-decorated CT26-derived extracellular vesicle (GLP@LTEV); 210.1-nm mean diameter; 90.0% GLP loading	BALB/c CT26 tumors about 60 mm³; GLP 90 mg/kg + LTEV 10 mg/kg i.v. on days 6, 9, 12, 15, and 18; efficacy n = 7/group; immune assays n = 3	PBS; LTEV; GLP	M1/M2 macrophages, dendritic-cell maturation, CD8-positive T cells, and regulatory T cells; formulation comparison without receptor knockout	Tumor inhibition: LTEV 24.0%, GLP 62.6%, GLP@LTEV 80.4%; CD11b+/CD86+ cells 45.4% vs. 20.2% with PBS; CD3+/CD8+ cells 14.0% vs. 1.9% with PBS	Small immune-assay sample; homotypic mouse-tumor-vesicle platform; no clinical exposure or safety data	56
*T. orientalis* TOP60-1; homogeneous heteropolysaccharide; 1.122 × 10^4^ g/mol	Tumor-cell assays and zebrafish xenograft model; doses as reported in the source	Untreated and assay-specific controls	TLR2/TLR4 interaction assays and CD40, CD80, CD86, and MHC-II macrophage markers	Dose-dependent inhibition of tumor growth, metastasis, angiogenesis, and migration	Zebrafish/cell evidence; no mammalian exposure bridge or clinical study	52
*G. frondosa* GFI-21alpha; homogeneous high-molecular-weight alpha-(1->4)-D-glucan with defined branch points	Breast-cancer cell assays and immunocompetent mouse tumor model; dosing as reported in the source	Vehicle and lentinan comparator	Intratumoral CD8-positive T cells and IFN-gamma-associated effector activity; direct cytotoxicity absent *in vitro*	Tumor suppression exceeded the lentinan comparator without overt toxicity in the reported experiment	Preclinical study; dose/exposure and causal receptor confirmation require clinical bridging	53
*G. lucidum* polysaccharide/chitosan-oligosaccharide full-cell nanovaccine (CGT)	Orthotopic, recurrent, and metastatic murine 4T1 models; dosing as reported in the source	Formulation and model-specific controls	Dendritic-cell targeting and maturation; antigen-specific immune responses	Inhibited primary tumor growth and distant lung metastasis in the reported mouse models	Engineered multicomponent vaccine; no human efficacy or formulation-equivalence evidence	55

BMDM, bone-marrow-derived macrophage; CAR-T, chimeric antigen receptor T cell; DC, dendritic cell; IFN, interferon; i.p., intraperitoneal; i.v., intravenous; LLC, Lewis lung carcinoma; MDSC, myeloid-derived suppressor cell; NK, natural killer; PBS, phosphate-buffered saline; TLR, Toll-like receptor. Exact dose units are stated where they differ from mg/kg. Significance thresholds and sample sizes are shown when reported and verified.

## 7 Oral delivery, gut immune interfaces, and microbiome hypotheses

Most marketed mushroom products are taken orally, whereas several historically developed glucans were administered parenterally. Demonstrated conventional systemic absorption of intact high-molecular-weight mushroom polysaccharides is limited. Gut-associated immune sampling, microbial fragmentation, barrier effects, and changes in metabolites are biologically plausible routes, but for most oncology preparations they remain supported preclinical mechanisms or hypotheses rather than established patient mechanisms (6, 34, 54).

An oral preparation may act locally without substantial intact polymer appearing in plasma. In an AOM/DSS colitis-associated tumorigenesis model, a water-soluble *Ganoderma lucidum* spore polysaccharide altered gut microbiota and short-chain-fatty-acid production, improved barrier measures, reduced inflammatory macrophage signals, and decreased tumor number and size at 200–300 mg/kg (34). This supports a microbiome-barrier-immune association in that specific mouse model; it does not demonstrate improved checkpoint-inhibitor response or an equivalent mechanism in patients. Diet, antibiotics, proton-pump inhibitors, chemotherapy, age, and geography may also change the biologically active exposure.

A 2026 AOM/DSS study further associated *Ganoderma lucidum* polysaccharide treatment with mitochondrial regulation, a higher M1/M2 macrophage ratio, and inhibition of colitis-associated colorectal-cancer progression; pharmacologic interference with mitochondrial fission attenuated the macrophage-polarization effect (65). This remains model-specific evidence and does not demonstrate a corresponding mechanism or benefit in patients.

The microbiome is especially relevant to cancer immunotherapy because intestinal communities can influence systemic immunity and treatment response. Nevertheless, showing that a mushroom polysaccharide changes stool composition is not evidence that it improves checkpoint-inhibitor efficacy. A demonstrated causal chain would require a reproducible microbial or metabolic change, association with a defined immune response, and transfer, depletion, or rescue evidence showing that the microbiome is necessary for the antitumor effect. In patients, baseline stool profiles, antibiotic exposure, diet, and concomitant medicines should be recorded prospectively and linked to pharmacodynamic and clinical endpoints (66).

Oral-route studies need direct measurements. Stable-isotope or fluorescent labeling can track intact polymer and fragments, although the label must remain associated with the relevant molecular species. Size-exclusion chromatography, mass spectrometry after controlled depolymerization, carbohydrate-specific assays, and tissue imaging can help distinguish absorption from degradation. Blood measurements alone may miss lymphatic transport or sequestration in liver and spleen. Sampling should therefore include time-resolved plasma or serum, urine and feces, and, where ethically feasible, tumor tissue, draining lymph nodes, or paired surgical specimens (27, 54).

The comparison between oral and parenteral routes should be made experimentally rather than assumed from tradition. Parenteral delivery may increase systemic exposure but can create infusion reactions, complement activation, or rapid clearance by the mononuclear phagocyte system. Oral delivery is convenient and may exploit mucosal pathways, but variability is likely to be greater. A dose-escalation program should define the route-specific pharmacokinetic and pharmacodynamic relationship, not simply administer grams of a supplement because that amount was previously consumed safely (27, 54).

This route problem helps explain why culinary intake, supplement use, and pharmaceutical glucan therapy cannot be treated as equivalent. Eating a mushroom exposes the patient to a food matrix containing proteins, fiber, lipids, minerals, and small molecules, with substantial cooking and digestive transformation. A purified or formulated candidate is produced to meet a molecular specification and may be delivered at a different dose and route. Nutritional epidemiology can generate hypotheses about long-term dietary exposure, but it cannot completely validate a preparation-specific immunotherapy claim (6, 27).

## 8 Human evidence: clinical outcomes versus immune surrogates

Human evidence is strongest for two historically developed products, PSK and lentinan, and substantially weaker for most edible-mushroom extracts. The studies are not interchangeable because products, routes, cancer types, standard treatments, and endpoints differ. Direct human evidence for mushroom-derived polysaccharides with checkpoint inhibitors, therapeutic antibodies, or cancer vaccines was not identified; contemporary combination evidence is primarily preclinical or derived from non-mushroom beta-glucans.

PSK has the most substantial randomized survival evidence among mushroom-derived preparations. In a multicenter trial after curative gastrectomy, Nakazato et al. (29) randomized 262 patients to standard mitomycin/fluorouracil treatment with or without PSK. At a minimum follow-up of five years, disease-free survival was 70.7% with PSK and 59.4% with standard treatment alone (p = 0.047); overall survival was 73.0% and 60.0%, respectively (p = 0.044). Toxicity was described as slight and similar between groups. These results are clinically meaningful, but the chemotherapy regimen and staging era differ from current gastric-cancer care, and the study does not establish that PSK would add benefit to modern perioperative chemotherapy, targeted therapy, or immunotherapy.

Ohwada et al. (30) evaluated PSK with oral tegafur/uracil after stage II or III colorectal-cancer resection. Of 207 registered patients, 205 were analyzed: 137 received PSK plus tegafur/uracil and 68 received tegafur/uracil alone. Five-year disease-free survival was 73.0% (95% CI 65.6%-80.4%) with PSK and 58.8% (95% CI 47.1%-70.5%) in controls (p = 0.016). Five-year overall survival was 81.8% versus 72.1% (p = 0.056), while the stage III subgroup showed larger differences in disease-free survival (60.0% vs. 32.1%, p = 0.002) and overall survival (74.6% vs. 46.4%, p = 0.003). The unequal 2:1 allocation, modest sample, subgroup interpretation, and dated comparator limit generalization.

Lentinan presents a more cautionary trajectory. An individual-patient-data meta-analysis assembled 650 patients from five older randomized trials of chemotherapy with or without lentinan for advanced gastric cancer and reported an overall-survival hazard ratio of 0.80 (95% CI 0.68-0.95) (67). The pooled result suggested benefit, but the trials were conducted across heterogeneous regimens and earlier standards of care. A later phase III trial provided a decisive contemporary test. Yoshino et al. (28) randomized 154 patients to S-1 alone and 155 to S-1 plus lentinan. Median overall survival was 13.8 and 9.9 months, respectively (p = 0.208); median time to treatment failure was 4.3 and 2.6 months (p < 0.001), and objective response rates were 22.3% and 18.7%. Adverse-event rates and quality of life were similar. The trial therefore found no overall-survival benefit and a significantly worse time to treatment failure with lentinan. A *post hoc* biomarker signal among patients with at least 2% lentinan-binding monocytes may not overturn the negative primary result and instead supports prospective biomarker testing.

Small studies of other preparations mainly show feasibility or immune modulation. Deng et al. (31) enrolled 34 postmenopausal breast-cancer survivors who were free of disease after initial treatment. Participants received an oral maitake liquid extract at 0.1, 0.5, 1.5, 3, or 5 mg/kg twice daily for three weeks. No dose-limiting toxicity occurred, although two participants withdrew with grade 1 symptoms. Across 146 immune measurements, 25 showed significant changes, and the dose-response pattern was non-monotonic: some functions increased while others decreased. This study is valuable because it challenges the simplistic idea of uniform “immune boosting,” but it was not designed to assess recurrence, tumor response, or survival.

Ahn et al. (38) studied 100 patients with cervical, ovarian, or endometrial cancer receiving carboplatin-based chemotherapy. Thirty-nine consumed an *Agaricus blazei* Murill Kyowa extract and 61 served as controls. NK-cell activity was higher in the extract group (p < 0.002), and selected chemotherapy-related symptoms improved, while lymphokine-activated killer-cell activity, monocyte activity, and immune-cell counts did not differ significantly. The lack of a tumor-response or survival endpoint, uncertainty around allocation and blinding, and the complex extract prevent an efficacy claim. The appropriate conclusion is that an adjunctive extract changed one immune function and patient-reported symptoms in a modest study.

Tangen et al. (39) randomized 40 patients with multiple myeloma undergoing high-dose chemotherapy and autologous stem-cell transplantation to an *Agaricus*-based extract (19 patients) or placebo (21 patients) for approximately seven weeks. Thirty-three were evaluable for the detailed immune endpoints. The extract group showed changes in regulatory T cells, plasmacytoid dendritic cells, serum interleukin-1 receptor antagonist, interleukin-5 and interleukin-7, and immune-gene expression. All 40 patients were included in survival analyses, but treatment response and overall survival did not differ significantly. Mean time to new treatment was 37.3 months with the extract and 31.4 months with placebo (p = 0.47). The intervention was a mixed preparation rather than a purified polysaccharide, so its strongest contribution is controlled evidence of immune modulation during intensive therapy, not proof of clinical efficacy.

Spacek et al. (37) followed 195 patients with stage I-II endocrine-dependent breast cancer in clinical and imaging remission; 49 received pleuran and 146 served as controls. The investigators reported increases in CD3-positive, CD19-positive, CD4-positive, and CD8-positive lymphocyte counts during follow-up. The study supports the feasibility of long-term immune monitoring but was nonrandomized, had markedly unequal group sizes, and did not establish a recurrence or survival benefit.

Taken together, the human literature supports four evidence-level conclusions. Demonstrated clinical outcomes are limited to particular historical PSK trials and the negative phase III lentinan result, not the class. Supported human biological activity consists mainly of preparation-specific immune-marker changes. Preliminary studies of maitake, *Agaricus*, and pleuran do not establish tumor control. Contemporary clinical efficacy, including benefit with checkpoint inhibitors, therapeutic antibodies, or cancer vaccines, remains untested or unproven for mushroom-derived preparations. No study justifies replacing established surgery, radiotherapy, systemic therapy, or approved immunotherapy with an edible mushroom, powder, tea, or unstandardized supplement (28–31, 37–39).

A recent review of mushroom-related clinical trials reached a similarly qualified picture: reported effects differed by mushroom component, dose, cancer type, and endpoint; breast-cancer survival signals were subgroup- or marker-specific, and colorectal-cancer survival findings were inconsistent (14). This contemporary synthesis does not supply direct clinical validation for mushroom-derived polysaccharides combined with checkpoint inhibitors, therapeutic antibodies, cancer vaccines, or CAR-T cells.

Future clinical trials should start with a pharmaceutical description of the tested material, not a species name. Investigators should publish the source, manufacturing process, structural fingerprint, purity, contaminant limits, stability, route, dose, and potency assay. Randomization should use a contemporary standard-care comparator, and the primary endpoint should match the claim: recurrence-free or overall survival for adjuvant efficacy, objective response or progression-free survival for advanced disease, validated symptom scales for supportive care, and prespecified immune biomarkers for mechanism. Exploratory cytokine panels should not replace a patient-centered endpoint. Tumor biopsies, when ethical and feasible, should test whether the intended myeloid or lymphoid change occurs in the tumor rather than only in peripheral blood (21, 27). The principal human studies and their limitations are summarized in **Table 4**.

**Table 4:** Human oncology evidence for mushroom-derived preparations.

Product and cancer setting	Design and sample size (n)	Intervention and comparator	Numerical outcomes (time units as reported)	Interpretation	References
PSK (polysaccharide-protein complex); gastric cancer	Randomized multicenter adjuvant trial; n = 262	Standard mitomycin/fluorouracil ± PSK after curative gastrectomy	5-year DFS 70.7% vs. 59.4% (p = 0.047); 5-year OS 73.0% vs. 60.0% (p = 0.044)	Positive patient outcomes, but historical regimen and staging	29
PSK (polysaccharide-protein complex); colorectal cancer	Randomized controlled trial; analyzed n = 205 (137 vs. 68)	UFT + PSK vs. UFT after stage II/III resection	5-year DFS 73.0% vs. 58.8% (p = 0.016); OS 81.8% vs. 72.1% (p = 0.056); stage III OS 74.6% vs. 46.4% (p = 0.003)	Unequal allocation, modest size, dated comparator, subgroup caution	30
Lentinan (purified beta-glucan); advanced gastric cancer	Randomized phase III; n = 309 (154 vs. 155)	S-1 vs. S-1 + lentinan	Median OS 13.8 vs. 9.9 months (p = 0.208); TTF 4.3 vs. 2.6 months (p < 0.001); ORR 22.3% vs. 18.7%	No efficacy; TTF significantly worse with lentinan	28
Maitake process-defined extract; breast-cancer survivors	Phase I/II dose escalation; n = 34	0.1–5 mg/kg orally twice daily for 3 weeks	No DLT; 2 grade-1 withdrawals; 25/146 immune parameters changed with non-monotonic dose effects	Not designed for tumor response, recurrence, or survival	31
*Agaricus* multicomponent extract; gynecologic cancers	Controlled adjunctive study; n = 100 (39 vs. 61)	Carboplatin-based chemotherapy with or without oral extract	Higher NK activity (p < 0.002); selected symptoms improved; no significant LAK/monocyte differences	Immune and symptom endpoints, not cancer efficacy	38
*Agaricus*-based multicomponent AndoSan; multiple myeloma	Randomized double-blind study; n = 40 (19 vs. 21)	High-dose chemotherapy/autologous transplant with extract or placebo	Immune-marker changes; mean time to new treatment 37.3 vs. 31.4 months (p = 0.47); no significant response or OS difference	Underpowered for clinical outcomes; mixed extract	39
Pleuran beta-glucan preparation; breast-cancer remission	Non-randomized study; n = 195 (49 vs. 146)	Long-term pleuran vs. control	Reported increases in CD3-, CD19-, CD4-, and CD8-positive lymphocyte counts during follow-up	Imbalanced, nonrandomized, no recurrence/survival proof	37

CI, confidence interval; DFS, disease-free survival; DLT, dose-limiting toxicity; LAK, lymphokine-activated killer; NK, natural killer; ORR objective response rate; OS, overall survival; TTF, time to treatment failure; UFT, tegafur/uracil. All p values are two-sided where reported by the source.

## 9 Delivery, pharmacokinetics, manufacturing, safety, and regulation

Pharmacokinetics is among the least developed parts of the field. High-molecular-weight polysaccharides are not handled like small molecules, and a reported oral dose does not reveal how much intact polymer or active fragment reaches blood, lymph, liver, spleen, or tumor. Candidate development should use labeled material, validated carbohydrate assays, size-resolved analysis, and mass-balance experiments to distinguish intact polymer, fragments, and metabolites. Measurements should be linked to receptor occupancy or another pharmacodynamic marker. Without exposure data, investigators cannot compare batches or routes and cannot distinguish biological failure from failed delivery (6, 27, 54).

Delivery engineering may address a measured limitation when it is hypothesis driven. *Ganoderma lucidum* polysaccharide-methotrexate conjugates, for example, have been assembled into pH-sensitive nanoparticles for preclinical co-delivery research (35). Such work supports formulation feasibility, not independent immunotherapy efficacy. Every particle or chemical derivative is a new product whose safety, pharmacokinetics, biodistribution, potency, particle size, charge, morphology, loading, release kinetics, protein corona, complement activation, uptake, and sterilization stability require separate evaluation.

Recent delivery studies support this product-specific approach. The 2025 *Ganoderma lucidum* polysaccharide-based full-cell nanovaccine and the 2026 polysaccharide-decorated extracellular-vesicle system improved tumor targeting or antitumor immune responses in mouse models, but each formulation introduced distinct carrier, conjugation, biodistribution, and release attributes (55, 56). A current lentinan review likewise identifies conjugation and nanoscale delivery as active research areas while noting the need for reproducible structure, pharmacokinetics, and clinical evaluation (13).

Manufacturing should be designed around critical quality attributes. The source strain, tissue, substrate, fermentation or cultivation conditions, extraction solvent, temperature, pH, time, fractionation, purification, drying, and storage should be controlled. Specifications should include identity, molecular-mass distribution, linkage or fingerprint data, protein content, residual nucleic acids, moisture, endotoxin, bioburden, mycotoxins, heavy metals, pesticides, and residual solvents as applicable. A validated potency assay should correlate with a mechanistic attribute and remain stable across production lots. Total polysaccharide content alone is inadequate for batch release (26, 27).

Safety cannot be inferred from culinary use when the product is concentrated, modified, or administered parenterally. Potential risks include allergy, gastrointestinal symptoms, immune activation, infusion reactions, complement effects, contamination, and interactions with anticancer or immunosuppressive medicines. A preparation that activates macrophages or cytokines may not be safe in a patient with autoimmune disease, severe infection, organ dysfunction, or checkpoint-inhibitor toxicity. Clinical protocols should define stopping rules, immune-related monitoring, concomitant medicines, and product accountability (27).

Regulatory classification depends on the product, route, intended use, claim, and jurisdiction. A therapeutic oncology claim requires a drug-development evidence package appropriate to the product, including chemistry and manufacturing controls, stability, validated assays, nonclinical safety, exposure characterization, and controlled clinical evidence. Food or supplement status does not itself establish therapeutic efficacy or permit extrapolation from a chemically different preparation (26, 27).

**Figure 1** integrates preparation attributes with receptor recognition, proximal signaling, myeloid and lymphoid responses, tumor-compartment effects, and potential combination strategies. The diagram separates mechanisms demonstrated mainly in general fungal or yeast beta-glucan systems from findings supported in selected mushroom-preparation models and from hypotheses that remain untested clinically.

**Figure 1:**
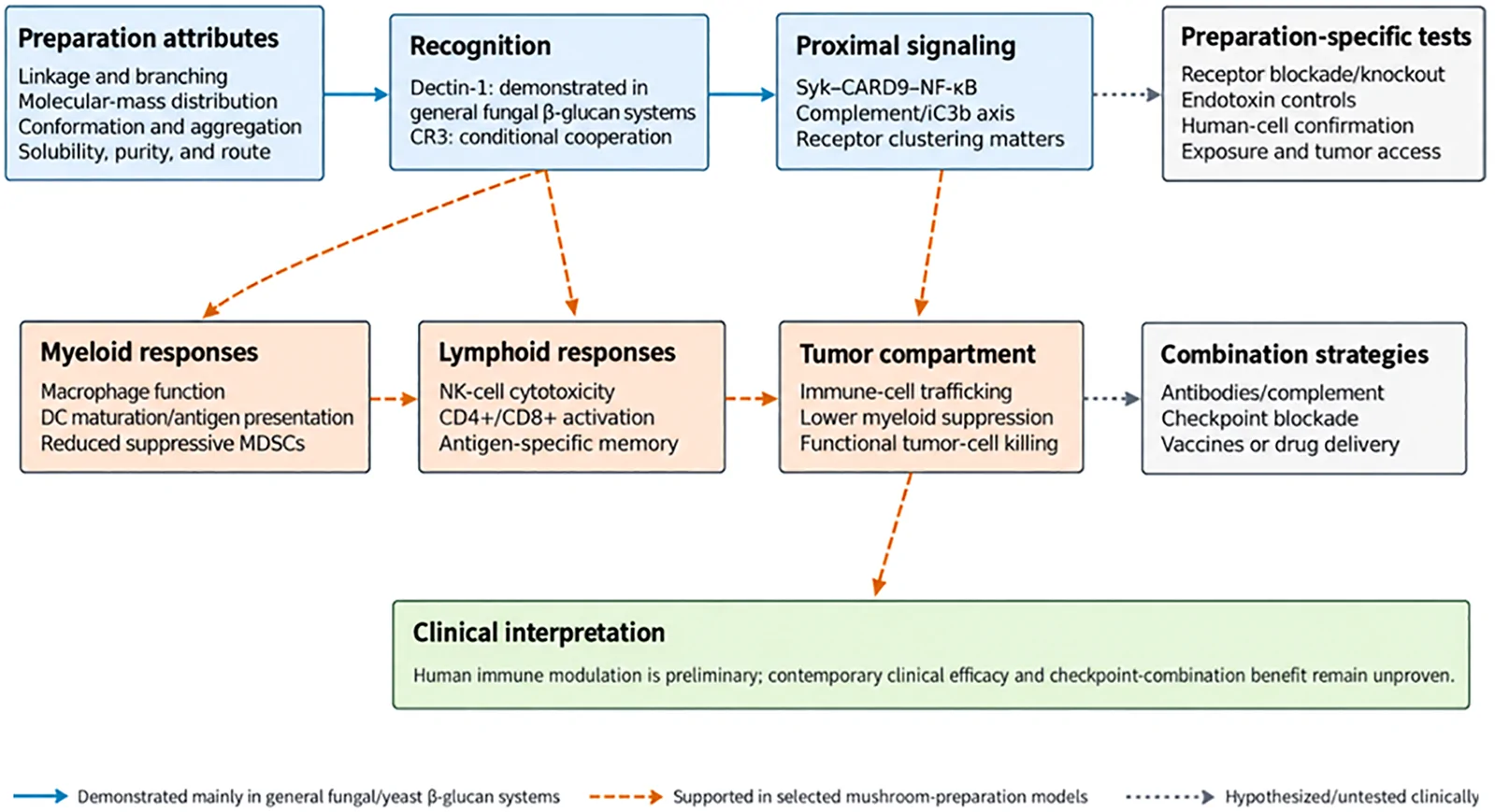
Mechanistic evidence map for mushroom-derived polysaccharide candidates. Solid blue arrows denote mechanisms demonstrated mainly in general fungal or yeast beta-glucan systems; dashed ochre arrows denote support from selected mushroom-preparation models; dotted gray arrows denote hypothesized links or clinically untested strategies. Product-specific receptor causality, route-specific exposure, and clinical combination benefit remain major gaps. The figure is a literature-informed synthesis and does not imply equal evidence strength across links. Evidence base: please arrange as : Brown et al. (16), Rogers et al. (17), Gross et al. (18), Goodridge et al. (19), Wang et al. (33), Taylor et al. (57), Hong et al. (58), LeibundGut-Landmann et al. (60), Wold et al. (25), Anaya et al. (49), Hu et al. (63), and Li et al. (32, 36).

The quality and reporting considerations in **Table 5** synthesize principles from botanical-drug and biologic specification guidance, animal-research and clinical-trial reporting standards, and the analytical literature on mushroom and beta-glucan preparations. They require adaptation to the product, route, development stage, jurisdiction, and intended claim (5, 7, 20, 21, 26, 27).

**Table 5:** Quality attributes for translational development.

Domain	Evidence-informed information to consider	Why it matters	References
Identity and provenance	Accepted species, strain/voucher, tissue, substrate, harvest, storage	Prevents species- and raw-material-level substitution	7, 27
Process definition	Extraction, pH, temperature, time, fractionation, purification, formulation	Makes batches and studies reproducible	7, 12, 26, 27
Structural specification	Molecular-mass distribution, monosaccharides, linkages, branching, conformation, protein content	Defines the candidate beyond total carbohydrate	5, 7, 11, 12, 26
Contaminant control	Endotoxin, bioburden, mycotoxins, metals, pesticides, residual solvents	Protects patients and prevents false immune activation	25–27
Mechanism-linked potency	Receptor-dependent reporter or functional immune assay with scientifically justified acceptance limits	Connects batch release to intended biology	16, 19, 26
Exposure and biodistribution	Validated assay for intact polymer/fragments, route-specific pharmacokinetics, tissue and tumor measurements	Distinguishes biological failure from failed delivery	13, 27, 54, 56
Preclinical translation	Immunocompetent models, established tumors, causal depletion/knockout tests, combination controls, toxicity	Reduces unsupported movement from cell assays to patients	2, 20
Clinical utility	Defined product, contemporary standard-care comparator, registration, biomarker, adverse events, patient endpoint	Permits interpretable benefit-risk assessment	21, 27

FDA, United States Food and Drug Administration; ICH, International Council for Harmonization. Requirements must be adapted to the product, route, development stage, jurisdiction, and intended claim.

## 10 Discussion

The literature supports biological plausibility but not a class-wide clinical claim. Demonstrated receptor-level evidence derives mainly from general fungal beta-glucan models of Dectin-1 clustering, Syk/CARD9 signaling, and conditional CR3 cooperation. Supported preparation-specific animal evidence includes tumor inhibition with myeloid remodeling or T-cell activation in selected *Grifola* and *Ganoderma* models. Preliminary human evidence shows immune modulation for some products, whereas demonstrated clinical outcomes are confined to specific historical PSK trials and a negative phase III lentinan trial. These evidence levels should not be collapsed into a single claim that mushroom polysaccharides treat cancer.

Product identity is the principal interpretive boundary. PSK, lentinan, pleuran, maitake fractions, *Ganoderma* polysaccharides, and *Agaricus* mixtures are chemically and pharmacologically different; positive results from one cannot validate another. Even within one named product, source strain, cultivation, manufacturing, formulation, dose, and route may change activity. Numerical outcomes should therefore remain attached to the exact preparation and experimental context.

A second controversy concerns the appropriate endpoint. Immune-marker changes may establish biological activity, but they are not inherently beneficial. Maitake produced bidirectional immune changes, and *Agaricus* studies changed NK activity or cytokines without proving tumor control. Future early-phase trials should select a small number of mechanistically justified biomarkers and show a consistent dose-exposure-response relationship. Later trials should test a patient outcome. A broad panel with several nominal p values and no correction can generate attractive but unstable signals.

The clinical context is also outdated. Most positive human outcome data involve older chemotherapy regimens, not checkpoint inhibitors, modern therapeutic antibodies, or cancer vaccines. Direct clinical evidence for contemporary immunotherapy combinations with mushroom-derived preparations remains a research gap. Future trials should identify a current unmet need and a rational partner, then enrich for the receptor, complement, or suppressive-myeloid context required by the proposed mechanism.

Recent studies include age-dependent *Agaricus blazei* polysaccharide activity after a 14-week preventive intervention in 8- and 12-month-old tumor-bearing mice, structurally defined alpha-glucan activity from *Grifola frondosa*, lentinan-enhanced CAR-T-cell activity, macrophage-directed *Ganoderma lucidum* polysaccharide effects, and engineered vaccine or extracellular-vesicle platforms (53, 55, 56, 64, 65, 68). None is a randomized human oncology trial, and the newer evidence should not be used to imply current clinical efficacy.

## 11 Evidence-informed translational priorities

The following roadmap is an evidence-informed proposal by the authors, not a universal regulatory requirement. Its elements should be adapted to the candidate’s route, intended claim, stage of development, jurisdiction, and applicable regulatory guidance. A target product profile should specify the cancer setting, combination partner, route, proposed mechanism, patient subgroup, pharmacodynamic marker, safety concerns, and clinical endpoint (20, 21, 26, 27).

Analytical qualification should compare independently manufactured batches using prespecified, scientifically justified ranges for molecular-mass distribution, monosaccharide composition, linkage fingerprint, branching, protein content, conformation or particle size where relevant, and contaminant limits. The number of batches and acceptance criteria should reflect product variability, development stage, intended use, and applicable guidance rather than a fixed universal minimum. Endotoxin control is essential in macrophage and dendritic-cell assays. A mechanism-linked potency assay should show concentration dependence, appropriate receptor interference where claimed, and discrimination between acceptable and deliberately degraded or structurally altered material. Analytical similarity should also be evaluated after formulation, sterilization, storage, and dilution into the administration vehicle (26, 27).

The second gate is human-mechanism confirmation. Experiments should include primary cells from healthy donors and from patients with the target cancer because chemotherapy, age, metabolic disease, and tumor-derived factors can alter receptor expression and immune responsiveness. The candidate should be tested in whole blood, purified immune populations, and, where possible, ex-vivo tumor cultures or organoids containing immune and stromal cells. Single-cell and spatial methods can identify responsive populations, but functional assays remain necessary. A transcriptional signature is useful only if it predicts phagocytosis, antigen presentation, T-cell activation, or tumor-cell killing.

The third gate is translational animal testing. The candidate should be administered at clinically feasible exposures and after tumors are established. At least one model should reproduce the proposed resistance mechanism, such as myeloid suppression, poor antigen presentation, or antibody-complement dependence. Monotherapy and combination arms are both needed. A combination claim requires evidence that the interaction is greater than either component alone and that the mechanism is lost after depletion of the relevant cell population or blockade of the receptor. Studies should report randomization, blinding where feasible, exclusions, individual tumor trajectories, body weight, hematology, serum chemistry, cytokines, and histopathology rather than presenting tumor volume alone (20).

An early clinical study should then focus on dose, exposure, safety, and target engagement. Escalation should seek an optimal biological dose, not automatically the maximum tolerated dose. The sampling plan should be defined before enrollment and should include baseline and on-treatment blood, with tumor tissue when clinically feasible. A small number of biomarkers should be chosen from the proposed mechanism. For a myeloid-directed candidate, these might include receptor occupancy, circulating and intratumoral suppressive myeloid cells, macrophage functional states, dendritic-cell activation, and antigen-specific T-cell responses. Cytokine panels should be interpreted alongside symptoms and organ toxicity because systemic inflammation is not synonymous with useful immunity.

A randomized efficacy-seeking study should be considered only after clinically feasible exposure, safety, and target engagement have been demonstrated; the phase and design should follow the development context and applicable guidance. The control group should receive the same contemporary standard care, and the clinical product should match the structural and potency specifications used in the supporting studies. The primary endpoint should match the intended claim: a patient-centered tumor or survival endpoint for efficacy, or a validated supportive-care endpoint when that is the stated objective. Immune biomarkers may be key secondary endpoints but should not substitute for clinical utility in a confirmatory study (21, 27).

Patient selection should follow the mechanism. A CR3-dependent strategy may require a tumor-targeting antibody capable of complement deposition and patients with adequate complement and effector-cell function. A myeloid-reprogramming candidate may be most useful in tumors with high suppressive-myeloid burden and measurable Dectin-1-pathway competence. A vaccine adjuvant requires a defined antigen and evidence that the candidate improves antigen-specific, not merely polyclonal, T-cell responses. Biomarker enrichment may reduce eligible numbers, but it is preferable to exposing a biologically unselected population to a low-probability intervention.

Statistical planning should account for the exploratory history of the field. Sample size should be based on a realistic effect size and attrition estimate, not the largest historical benefit. Randomization should be stratified by major prognostic factors, multiplicity controlled when several biomarkers or subgroups are tested, and analyses conducted according to a prespecified plan with transparent reporting of missing data and deviations (21).

Safety assessment should be product and combination specific. Monitoring should include immediate hypersensitivity or infusion reactions, inflammatory syndromes, hepatic and renal injury, hematologic effects, gastrointestinal intolerance, infection, and interactions with chemotherapy, antibodies, cellular therapies, or checkpoint inhibitors. Products intended for immunocompromised patients require strict microbial and mycotoxin control. Because immune stimulation can theoretically worsen autoimmune or inflammatory disease, exclusion criteria and stopping rules should be justified rather than copied from dietary-supplement studies (27).

Reproducibility should be planned from the start. Reference samples, spectra, chromatograms, molecular-mass data, and the potency-assay protocol should be archived. Trial batches should be traceable to strain, substrate, harvest, processing, and storage records. Raw numerical data and analysis code should be shared where consent and regulation permit. Independent replication with a separately manufactured batch is particularly important for a complex macromolecule. A result that depends on one inaccessible commercial lot is not a durable clinical finding (20, 26, 27).

This blueprint also defines rational stopping points. Development should pause when the product cannot be chemically reproduced, when apparent immune activity disappears after endotoxin control, when clinically feasible exposure does not engage the target, when the mechanism is absent in human tumor tissue, or when a randomized signal is weaker than toxicity or treatment burden. Stopping an unsuitable candidate is a scientific success because it protects patients and directs resources toward better-defined products.

## 12 Conclusion

The conclusions differ by evidence level. At the receptor level, Dectin-1/Syk/CARD9 signaling, ligand-presentation effects, and conditional CR3 cooperation are demonstrated mainly in general fungal or yeast beta-glucan systems; they require direct confirmation for each mushroom preparation. At the animal level, selected *Grifola* and *Ganoderma* preparations support antitumor effects linked to myeloid remodeling or T-cell activity, but characterization, route, exposure, and model limitations remain. At the human biological level, small studies support preliminary preparation-specific immune modulation without proving tumor control. At the clinical-efficacy level, historical PSK benefits are context-specific, the phase III lentinan trial was negative for overall survival, and contemporary combination benefit with checkpoint inhibitors, therapeutic antibodies, or cancer vaccines is not established. Mushroom-derived polysaccharides therefore do not constitute a clinically validated therapeutic class. Future candidates should be developed as reproducible products with causal mechanisms, route-specific pharmacokinetics, tumor pharmacodynamics, safety controls, and randomized comparison with contemporary standard care. Mushroom foods and unstandardized supplements should not substitute for established cancer treatment.

## References

[1] Sung H Filho AM Laversanne M Ferlay J Siegel RL Soerjomataram I . Global cancer statistics 2024: GLOBOCAN estimates of incidence and mortality worldwide for 34 cancers in 186 countries. CA: A Cancer J For Clin. (2026) 76:e70090. doi: 10.3322/caac.70090 42417444 PMC13343830

[2] Hanahan D . Hallmarks of cancer: New dimensions. Cancer Discov. (2022) 12:31–46. doi: 10.1158/2159-8290.CD-21-1059 35022204

[3] Spranger S Bao R Gajewski TF . Melanoma-intrinsic beta-catenin signalling prevents anti-tumour immunity. Nature. (2015) 523:231–5. doi: 10.1038/nature14404 25970248

[4] Zaretsky JM Garcia-Diaz A Shin DS Escuin-Ordinas H Hugo W Hu-Lieskovan S . Mutations associated with acquired resistance to PD-1 blockade in melanoma. N Engl J Med. (2016) 375:819–29. doi: 10.1056/NEJMoa1604958 27433843 PMC5007206

[5] Han B Baruah SK Cox E Vanrompay D Bossier P . Structure-functional activity relationship of beta-glucans from the perspective of immunomodulation: A mini-review. Front Immunol. (2020) 11:658. doi: 10.3389/fimmu.2020.00658 32391005 PMC7188827

[6] Cerletti C Esposito S Iacoviello L . Edible mushrooms and beta-glucans: Impact on human health. Nutrients. (2021) 13:2195. doi: 10.3390/nu13072195 34202377 PMC8308413

[7] Dilrukshi N Grice ID Mallard B Tiralongo J . Mushroom beta-glucans as immunomodulators: Elucidation of structure-function relationship. Food Chem. (2025) 495:146468. doi: 10.1016/j.foodchem.2025.146468 41187460

[8] Sivanesan I Muthu M Gopal J Oh J-W . Mushroom polysaccharide-assisted anticarcinogenic mycotherapy: Reviewing its clinical trials. Molecules. (2022) 27:4090. doi: 10.3390/molecules27134090 35807336 PMC9267963

[9] Noorbakhsh Varnosfaderani SM Ebrahimzadeh F Akbari Oryani M Khalili S Almasi F Mosaddeghi Heris R . Potential promising anticancer applications of beta-glucans: A review. Biosci Rep. (2024) 44:BSR20231686. doi: 10.1042/BSR20231686 38088444 PMC10776902

[10] Górska-Jakubowska S Wu Y Turło J Xu B . Critical review on the anti-tumor activity of bioactive compounds from edible and medicinal mushrooms over the last five years. Nutrients. (2025) 17:1887. doi: 10.3390/nu17111887 40507156 PMC12157108

[11] Gao Z Liu X Yu J Li Z Shi H Zhang G . Structural basis of immunomodulation by edible fungal polysaccharides: From molecular characteristics to action mechanisms. Carbohydr Res. (2025) 555:109591. doi: 10.1016/j.carres.2025.109591 40592243

[12] Li C Zhang R Tian B Liu B Mei Y Li W . A review of the extraction technologies, structural characterization, chemical modification, and pharmacological effects of *Ganoderma lucidum* polysaccharides. Naunyn-Schmiedeberg's Arch Pharmacol. (2025) 398:16967–98. doi: 10.1007/s00210-025-04436-w 40719895

[13] Zhen C Guo J Li R Xiao H Yang J Wang L . Comprehensive mechanisms and advanced delivery strategies of lentinan in antitumor therapy: A review. Colloids Surfaces B Biointerfaces. (2026) 258:115228. doi: 10.1016/j.colsurfb.2025.115228 41197335

[14] Bukovics MP Simon-Szabó L Takács I Németh Z . Beta-glucans, triterpenes and nucleoside analogs of edible and medicinal mushrooms as complementary treatment in diabetes and cancer-a review of evidence from clinical trials. Cancers. (2026) 18:2294. doi: 10.3390/cancers18142294 42512356 PMC13407415

[15] Kerrigan RW . *Agaricus subrufescens*, a cultivated edible and medicinal mushroom, and its synonyms. Mycologia. (2005) 97:12–24. doi: 10.3852/mycologia.97.1.12 16389952

[16] Brown GD Taylor PR Reid DM Willment JA Williams DL Martinez-Pomares L . Dectin-1 is a major beta-glucan receptor on macrophages. J Exp Med. (2002) 196:407–12. doi: 10.1084/jem.20020470 12163569 PMC2193936

[17] Rogers NC Slack EC Edwards AD Nolte MA Schulz O Schweighoffer E . Syk-dependent cytokine induction by Dectin-1 reveals a novel pattern recognition pathway for C type lectins. Immunity. (2005) 22:507–17. doi: 10.1016/j.immuni.2005.03.004 15845454

[18] Gross O Gewies A Finger K Schafer M Sparwasser T Peschel C . Card9 controls a non-TLR signalling pathway for innate anti-fungal immunity. Nature. (2006) 442:651–6. doi: 10.1038/nature04926 16862125

[19] Goodridge HS Reyes CN Becker CA Katsumoto TR Ma J Wolf AJ . Activation of the innate immune receptor Dectin-1 upon formation of a phagocytic synapse. Nature. (2011) 472:471–5. doi: 10.1038/nature10071 21525931 PMC3084546

[20] Percie du Sert N Hurst V Ahluwalia A Alam S Avey MT Baker M . The ARRIVE guidelines 2.0: Updated guidelines for reporting animal research. PloS Biol. (2020) 18:e3000410. doi: 10.1371/journal.pbio.3000410 32663219 PMC7360023

[21] Schulz KF Altman DG Moher D CONSORT Group . CONSORT 2010 Statement: Updated guidelines for reporting parallel group randomised trials. BMJ. (2010) 340:c332. doi: 10.1136/bmj.c332 20332509 PMC2844940

[22] Chihara G Maeda Y Hamuro J Sasaki T Fukuoka F . Inhibition of mouse sarcoma 180 by polysaccharides from *Lentinus edodes* (Berk.) Sing. Nature. (1969) 222:687–8. doi: 10.1038/222687a0 5768289

[23] Karacsonyi S Kuniak L . Polysaccharides of *Pleurotus ostreatus*: Isolation and structure of pleuran, an alkali-insoluble beta-D-glucan. Carbohydr Polym. (1994) 24:107–11. doi: 10.1016/0144-8617(94)90019-1

[24] Nanba H Hamaguchi A Kuroda H . The chemical structure of an antitumor polysaccharide in fruit bodies of *Grifola frondosa* (maitake). Chem Pharm Bull. (1987) 35:1162–8. doi: 10.1248/cpb.35.1162 3607939

[25] Wold C. W. Christopoulos P. F. Arias M. A. Dzovor D. E. Øynebråten I. Corthay A. . Fungal polysaccharides from *Inonotus obliquus* are agonists for Toll-like receptors and induce macrophage anti-cancer activity. Commun Biol. (2024) 7:222. doi: 10.1038/s42003-024-05853-y 38396285 PMC10891174

[26] International Conference for Harmonisation of Technical Requirements for Pharmaceuticals for Human Use (ICH) . Q6B: specifications: test procedures and acceptance criteria for biotechnological/biological products. In: Ich (1999).

[27] Food and Drug Administration (FDA) . Botanical drug development: guidance for industry. Revision 1. In: U.s. Department of Health and Human Services, Food and Drug Administration, Center for Drug Evaluation and Research (2016).

[28] Yoshino S Nishikawa K Morita S Takahashi T Sakata K Nagao J . Randomised phase III study of S-1 alone versus S-1 plus lentinan for unresectable or recurrent gastric cancer (JFMC36-0701). Eur J Cancer. (2016) 65:164–71. doi: 10.1016/j.ejca.2016.06.012 27501505

[29] Nakazato H Koike A Saji S Ogawa N Sakamoto J . Efficacy of immunochemotherapy as adjuvant treatment after curative resection of gastric cancer. Lancet. (1994) 343:1122–6. doi: 10.1016/S0140-6736(94)90233-X 7910230

[30] Ohwada S Ikeya T Yokomori T Kusaba T Roppongi T Takahashi T . Adjuvant immunochemotherapy with oral tegafur/uracil plus PSK in patients with stage II or III colorectal cancer: A randomised controlled study. Br J Cancer. (2004) 90:1003–10. doi: 10.1038/sj.bjc.6601619 14997197 PMC2409633

[31] Deng G Lin H Seidman A Fornier M D'Andrea G Wesa K . A phase I/II trial of a polysaccharide extract from *Grifola frondosa* (Maitake mushroom) in breast cancer patients: Immunological effects. J Cancer Res Clin Oncol. (2009) 135:1215–21. doi: 10.1007/s00432-009-0562-z 19253021 PMC3751581

[32] Li X Ruan Q Yang W Tian H Wu N Qadir J . Polysaccharide isolated from *Grifola frondosa* eliminates myeloid-derived suppressor cells and inhibits tumor growth by enhancing T cell responses. Int J Biol Sci. (2024) 20:664–79. doi: 10.7150/ijbs.85276 38169590 PMC10758108

[33] Wang Y Fan X Wu X . *Ganoderma lucidum* polysaccharide enhances antitumor immune response by regulating differentiation and inhibition of myeloid-derived suppressor cells via a CARD9-NF-kappaB-IDO pathway. Biosci Rep. (2020) 40:BSR20201170. doi: 10.1042/BSR20201170 32530032 PMC7313449

[34] Guo C Guo D Fang L Sang T Wu J Guo C . *Ganoderma lucidum* polysaccharide modulates gut microbiota and immune cell function to inhibit inflammation and tumorigenesis in colon. Carbohydr Polym. (2021) 267:118231. doi: 10.1016/j.carbpol.2021.118231 34119183

[35] Zheng D Zhao J Li Y Zhu L Jin M Wang L . Self-assembled pH-sensitive nanoparticles based on *Ganoderma lucidum* polysaccharide-methotrexate conjugates for the co-delivery of anti-tumor drugs. ACS Biomaterials Sci Eng. (2021) 7:3764–73. doi: 10.1021/acsbiomaterials.1c00663 34213326

[36] Li W Zhou Q Lv B Li N Bian X Chen L . *Ganoderma lucidum* polysaccharide supplementation significantly activates T-cell-mediated antitumor immunity and enhances anti-PD-1 immunotherapy efficacy in colorectal cancer. J Agric Food Chem. (2024) 72:12072–82. doi: 10.1021/acs.jafc.3c08385 38750669

[37] Spacek J Vocka M Zavadova E Konopasek B Petruzelka L . Immunomodulation with beta-glucan from *Pleurotus ostreatus* in patients with endocrine-dependent breast cancer. Immunotherapy. (2022) 14:31–40. doi: 10.2217/imt-2021-0069 34784798

[38] Ahn W-S Kim D-J Chae G-T Lee J-M Bae S-M Sin J-I . Natural killer cell activity and quality of life were improved by consumption of a mushroom extract, *Agaricus blazei* Murill Kyowa, in gynecological cancer patients undergoing chemotherapy. Int J Gynecological Cancer. (2004) 14:589–94. doi: 10.1111/j.1048-891X.2004.14403.x 15304151

[39] Tangen J-M Tierens A Caers J Binsfeld M Olstad OK Troseid A-MS . Immunomodulatory effects of the *Agaricus blazei* Murrill-based mushroom extract AndoSan in patients with multiple myeloma undergoing high-dose chemotherapy and autologous stem cell transplantation: A randomized, double-blinded clinical study. BioMed Res Int. (2015) 2015:718539. doi: 10.1155/2015/718539 25664323 PMC4312620

[40] Ren Z Qin T Qiu F Song Y Lin D Ma Y . Immunomodulatory effects of hydroxyethylated *Hericium erinaceus* polysaccharide on macrophages RAW264.7. Int J Biol Macromol. (2017) 105:879–85. doi: 10.1016/j.ijbiomac.2017.07.104 28729219

[41] Yang Y Li J Hong Q Zhang X Liu Z Zhang T . Polysaccharides from *Hericium erinaceus* fruiting bodies: Structural characterization, immunomodulatory activity and mechanism. Nutrients. (2022) 14:3721. doi: 10.3390/nu14183721 36145096 PMC9503163

[42] Jia W Feng J Zhang J-S Lin C-C Wang W-H Chen H-G . Structural characteristics of the novel polysaccharide FVPA1 from winter culinary-medicinal mushroom, *Flammulina velutipes*, capable of enhancing natural killer cell activity against K562 tumor cells. Int J Med Mushrooms. (2017) 19:535–46. doi: 10.1615/IntJMedMushrooms.v19.i6.50 29199563

[43] Wang W-H Zhang J-S Feng T Deng J Lin C-C Fan H . Structural elucidation of a polysaccharide from *Flammulina velutipes* and its immunomodulation activities on mouse B lymphocytes. Sci Rep. (2018) 8:3120. doi: 10.1038/s41598-018-21375-0 29449636 PMC5814432

[44] Misaki A Kakuta M Sasaki T Tanaka M Miyaji H . Studies on interrelation of structure and antitumor effects of polysaccharides: Antitumor action of periodate-modified, branched (1,3)-beta-D-glucan of *Auricularia auricula-judae*, and other polysaccharides containing (1,3)-glycosidic linkages. Carbohydr Res. (1981) 92:115–29. doi: 10.1016/s0008-6215(00)85986-8 7196285

[45] Nilkhet S Mongkolpobsin K Sillapachaiyaporn C Wongsirojkul N Tencomnao T Chuchawankul S . M1 macrophages polarized by crude polysaccharides isolated from *Auricularia polytricha* exhibit anti-tumor effect on human breast cancer cells. Sci Rep. (2024) 14:8179. doi: 10.1038/s41598-024-58208-2 38589471 PMC11001921

[46] Zhao J Xie W Liu X Zeng Y Jin X Li G . A novel acidic polysaccharide from *Inonotus obliquus* (IOP-1) suppresses growth and induces apoptosis in non-small cell lung cancer both *in vitro* and *in vivo*. Curr Med Chem. (2026). doi: 10.2174/0109298673471395260513145149 42283192. Advance online publication.

[47] Zhou X Zhang L Hu W Liu X Song S Peng S . Characterization and antitumor activities of polysaccharides derived from *Inonotus sanghuang* against cancers of the liver and lungs. Int J Biol Macromol. (2026) 354:151359. doi: 10.1016/j.ijbiomac.2026.151359 41819334

[48] Guo H Wei B Wang Z Liu L Normakhamatov N . Growth inhibition of hepatocellular carcinoma by the polysaccharide-protein complex from *Inonotus hispidus* mycelia and the mechanisms. J Sci Food Agric. (2026). doi: 10.1002/jsfa.70970 42596063

[49] Anaya EU Etemadi Amin A Wester MJ Danielson ME Michel KS Neumann AK . Dectin-1 multimerization and signaling depends on fungal beta-glucan structure and exposure. Biophys J. (2023) 122:3749–67. doi: 10.1016/j.bpj.2023.07.021 37515324 PMC10541497

[50] Zhang Q Du Z Zhang Y Zheng Z Li Q Wang K . Apoptosis induction activity of polysaccharide from *Lentinus edodes* in H22-bearing mice through ROS-mediated mitochondrial pathway and inhibition of tubulin polymerization. Food Nutr Res. (2020) 64:4364. doi: 10.29219/fnr.v64.4364 33240031 PMC7672475

[51] Feng M Fu F Lei M Luo W Su Y Xie Y . Structural feature and antitumor activity of a N-acetylglucosamine containing polysaccharide from *Ganoderma sinense*. Carbohydr Polym. (2025) 366:123838. doi: 10.1016/j.carbpol.2025.123838 40733790

[52] He W Jiang H Rong M Li X Xu J Guo Y . A fungal polysaccharide from *Trametes orientalis*: Structural elucidation and multifaceted antitumor mechanisms via metastasis, angiogenesis, and immunomodulation. Carbohydr Polym. (2025) 368:124151. doi: 10.1016/j.carbpol.2025.124151 40947190

[53] Tian HX Ruan QZ Xie YZ Chen W Xiao C Zhong W . Structural characterization and antitumor activity via immune modulation of an alpha-glucan from *Grifola frondosa*. Carbohydr Polym. (2026) 382:125254. doi: 10.1016/j.carbpol.2026.125254 42002344

[54] De Marco Castro E Calder PC Roche HM . Beta-1,3/1,6-glucans and immunity: State of the art and future directions. Mol Nutr Food Res. (2021) 65:1901071. doi: 10.1002/mnfr.201901071 32223047 PMC7816268

[55] Hu W Ma Y Zhang Q Zhang Q Yu X Wang H . DCs-targeted full-cell nanovaccine based on chitosan oligosaccharide self-assembled with *Ganoderma lucidum* polysaccharide for enhancing tumor immunotherapy. Int J Biol Macromol. (2025) 334:148934. doi: 10.1016/j.ijbiomac.2025.148934 41253106

[56] Ouyang Z Li S Zhang A Ye S Ye W Wen G . *Ganoderma lucidum* polysaccharide-decorated extracellular vesicle enables synergistical antitumor immunotherapy. Mater Today Bio. (2026) 37:102832. doi: 10.1016/j.mtbio.2026.102832 41640603 PMC12864664

[57] Taylor PR Brown GD Reid DM Willment JA Martinez-Pomares L Gordon S . The beta-glucan receptor, Dectin-1, is predominantly expressed on the surface of cells of the monocyte/macrophage and neutrophil lineages. J Immunol. (2002) 169:3876–82. doi: 10.4049/jimmunol.169.7.3876 12244185

[58] Hong F Hansen RD Yan J Allendorf DJ Baran JT Ostroff GR . Beta-glucan functions as an adjuvant for monoclonal antibody immunotherapy by recruiting tumoricidal granulocytes as killer cells. Cancer Res. (2003) 63:9023–31. 14695221

[59] Wang W-J Wu Y-S Chen S Liu C-F Chen S-N . Mushroom beta-glucan may immunomodulate the tumor-associated macrophages in the Lewis lung carcinoma. BioMed Res Int. (2015) 2015:604385. doi: 10.1155/2015/604385 26167490 PMC4488085

[60] LeibundGut-Landmann S Gross O Robinson MJ Osorio F Slack EC Tsoni SV . Syk- and CARD9-dependent coupling of innate immunity to the induction of T helper cells that produce interleukin 17. Nat Immunol. (2007) 8:630–8. doi: 10.1038/ni1460 17450144

[61] Smiderle FR Ruthes AC van Arkel J Chanput W Iacomini M Wichers HJ . Polysaccharides from *Agaricus bisporus* and *Agaricus brasiliensis* show similarities in their structures and their immunomodulatory effects on human monocytic THP-1 cells. BMC Complementary Altern Med. (2011) 11:58. doi: 10.1186/1472-6882-11-58 21787425 PMC3158557

[62] Wang M Bai Y Pei J Li D Pu X Zhu W . Beta-glucan combined with PD-1/PD-L1 checkpoint blockade for immunotherapy in patients with advanced cancer. Front Pharmacol. (2022) 13:887457. doi: 10.3389/fphar.2022.887457 35548349 PMC9084312

[63] Hu X Shui Y Hirano H Kusano K Guo W-Z Fujino M . PD-L1 antibody enhanced beta-glucan antitumor effects via blockade of immune checkpoints in a melanoma model. Cancer Immunol Immunother. (2023) 72:719–31. doi: 10.1007/s00262-022-03276-4 36053290 PMC10992518

[64] Niu X Zhang P Liu Z Tang Y Xu S Wan X . Lentinan enhances CAR-T cell potency in solid tumors by optimizing T cell differentiation. Front Immunol. (2025) 16:1605488. doi: 10.3389/fimmu.2025.1605488 40755764 PMC12313588

[65] Ouyang Z Ye W Wen G Li S Wei T Chen M . The role of mitochondrial regulation in macrophage polarization by *Ganoderma lucidum* polysaccharide for the treatment of colitis-associated colorectal cancer. Mol Cell Biochem. (2026) 481:3071–84. doi: 10.1007/s11010-026-05584-0 42228269

[66] Gopalakrishnan V Spencer CN Nezi L Reuben A Andrews MC Karpinets TV . Gut microbiome modulates response to anti-PD-1 immunotherapy in melanoma patients. Science. (2018) 359:97–103. doi: 10.1126/science.aan4236 29097493 PMC5827966

[67] Oba K Kobayashi M Matsui T Kodera Y Sakamoto J . Individual patient based meta-analysis of lentinan for unresectable/recurrent gastric cancer. Anticancer Res. (2009) 29:2739–45. 19596954

[68] Xu Y Xu T Huang C Amakye WK Li K Zhu Y . The age-dependent anticancer efficacy of *Agaricus blazei* Murill polysaccharide in colon cancer. Mol Nutr Food Res. (2025) 69:e202400733. doi: 10.1002/mnfr.202400733 39950425

